# How Necessary is the Vasculature in the Life of Neural Stem and Progenitor Cells? Evidence from Evolution, Development and the Adult Nervous System

**DOI:** 10.3389/fncel.2016.00035

**Published:** 2016-02-16

**Authors:** Christos Koutsakis, Ilias Kazanis

**Affiliations:** ^1^Laboratory of Developmental Biology, Department of Biology, University of PatrasPatras, Greece; ^2^Wellcome Trust-MRC Cambridge Stem Cell Institute, University of CambridgeCambridge, UK

**Keywords:** neural stem cells, vasculature, blood vessels, neurogenesis, proliferation, differentiation, migration, stem cell niche

## Abstract

Augmenting evidence suggests that such is the functional dependance of neural stem cells (NSCs) on the vasculature that they normally reside in “perivascular niches”. Two examples are the “neurovascular” and the “oligovascular” niches of the adult brain, which comprise specialized microenvironments where NSCs or oligodendrocyte progenitor cells survive and remain mitotically active in close proximity to blood vessels (BVs). The often observed co-ordination of angiogenesis and neurogenesis led to these processes being described as “coupled”. Here, we adopt an evo-devo approach to argue that some stages in the life of a NSC, such as specification and commitment, are independent of the vasculature, while stages such as proliferation and migration are largely dependent on BVs. We also explore available evidence on the possible involvement of the vasculature in other phenomena such as the diversification of NSCs during evolution and we provide original data on the senescence of NSCs in the subependymal zone stem cell niche. Finally, we will comment on the other side of the story; that is, on how much the vasculature is dependent on NSCs and their progeny.

## Introduction

The observation that neurogenesis and angiogenesis are seasonally coordinated in the brain of songbirds (Louissaint et al., 2002) produced the first evidence on the existence of a cross-talk between neural stem cells (NSCs) and blood vessels (BVs). More recently, it was shown that endothelial cells control the function of adult brain NSCs via direct cell contact and diffusible signals (Ottone and Parrinello, 2015). But is this the truth and nothing but the truth? The first neurons and glia appeared in animals that had no vasculature (Satterlie, 2015) and in early neurodevelopmental stages of mammals NSCs emerge and form the neural tube in the absence of vascularization. This strongly suggests that NSCs can exist and function in the absence of BVs and raises the challenging question: how much does the existence and the function of NSCs depend on the vasculature?

**Figure 1:**
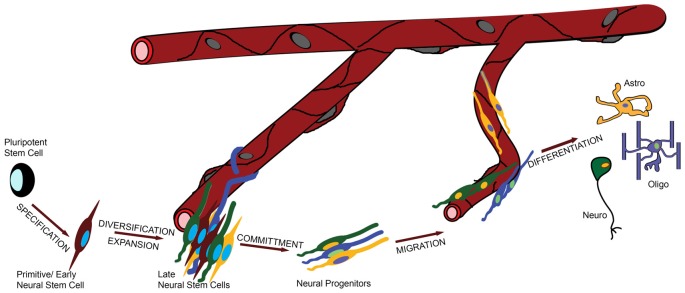
**The role of the vasculature in the life of a neural stem cell (NSC).** In this graphic illustration, the different stages in the life of a NSC are shown and the involvement of the vasculature is depicted by the distance of the cells from the blood vessels (BVs). For example, specification and commitment of NSCs appear to happen away from the vessels, whilst proliferation and migration in close proximity.

To address this question in a systematic and comprehensive way we defined the major functional stages in the life of a NSC, informed both by evolution and development (Figure 1): (i) specification of NSC identity from earlier pluripotent—e.g., embryonic or ancestral stem cells; (ii) proliferation/diversification, during which the pool of NSCs is expanded and becomes heterogeneous; (iii) commitment to a specific fate (such as neuronal or glial); (iv) migration and finally; (v) differentiation. During stage (iii) NSCs become, or give rise to, neural progenitors, and overall migration can be minimal or absent.

## Generalizations

### The *In Vitro* Life of NSCs

Neural stem cell culture protocols (iPSCs, primary cells, cell lines) have proven that all stages in the life of a NSC can be recapitulated *in vitro*. Embryonic or induced stem cells can be programmed to adopt NSC fate and can be differentiated into a range of neuronal and glial cell types. This, obviously, does not exclude the possibility that BVs or their ancestral systems are necessary *in vivo*, especially as cell culture media are rich in components that are provided by BVs in the tissue. Endothelial cells have been found to enhance neurogenesis in many cell culture assays, but few studies have gone the extra mile to directly link these *in vitro* results to the role of endothelial cells in the live organism (Shen et al., 2004; Androutsellis-Theotokis et al., 2010).

### Main Mechanisms of NSC-BV Interaction

NSC-BV interaction can be achieved through three different, but possibly co-operating, mechanisms. First, via direct contact between NSCs and BV components, such as endothelial cells and perivascular extracellular matrix (Javaherian and Kriegstein, 2009; Ottone et al., 2014). Second, via diffusible signals generated by vascular and perivascular cells, such as in the case of endothelium-derived neurotrophin-3 (Delgado et al., 2014). Third, via diffusible signals that BVs transport but don’t generate themselves. In small organisms, such as planarians, nutrients can be diffused directly from the environment, and in insects NSCs are directly bathed in the hemeolymph (Limmer et al., 2014; Spéder and Brand, 2014). In larger animals blood circulation is required for necessary factors to reach their target areas. One such example, crucial for NSCs, is insulin (Masjkur et al., 2012). Recently we have identified a possible fourth mechanism, in which the function of NSCs is controlled by platelets (a circulatory element), possibly via active mediation by endothelial cells (elements of the BV structure; Kazanis et al., 2015).

### What is the Nature of a NSC?

It remains challenging to define what a NSC is and when it is reduced to neural progenitor status (exhibiting a more restricted potential). Here, we adopt an extended version of the unified hypothesis (Alvarez-Buylla et al., 2001), according to which the cardinal NSC properties are found equally in primitive/early NSCs with a neuroepithelial-like phenotype, in more developed cells with a radial glial phenotype and in some species (mostly in mammals), in mature cells with an astroglial phenotype (Figure 1). A surprising deviation was recently reported in the adult crayfish brain, in which the neurogenic stem cell pool does not contain *bona fide* NSCs but is constantly replenished from the hematopoietic system. Vascular extensions of the cerebral artery facilitate this process and this is an intriguing example of vessel-dependent support of neurogenesis (Chaves da Silva et al., 2013; Benton et al., 2014).

## The Role of the Vasculature

### Specification

The *in vitro* (e.g., from iPSC differentiation or trans-differentiation experiments) and *in vivo* (e.g., from early embryonic developmental stages, or from evolutionary evidence based on zoological observations) data currently available indicate that the specification of pluripotent stem cells towards a neural identity does not depend on any form of interaction with some type of vasculature. The first neuron-like sensory cells and primitive nervous systems appeared in species that lacked BVs (Jékely et al., 2015) and the specification of neuroectoderm and of neuroepithelial cells in mammals marginally precedes the angiogenic specification of mesoderm in the forebrain (Vasudevan et al., 2008; Javaherian and Kriegstein, 2009). Nevertheless, after the closure of the neural tube, the primitive neuroepithelium remains in contact with a CSF-like fluid that is partly constituted by the early BV network of deeper layers (Lun et al., 2015).

### Proliferation/Diversification

As soon as NSCs become specified the processes of self-renewal and expansion are initiated. Low levels of proliferation, adequate for the generation of single neurons and glia and for the construction of primitive neuronal networks and of the early neural tube, can occur in the absence of vascularization (Rodriguez Celin et al., 2015). The emergence of larger and more complicated nervous systems required higher levels of proliferation in embryonic neural stem and progenitor cells, and this was partly achieved through their diversification. The evolution of the neocortex was facilitated by the appearance of radial glial-type NSCs (see “What is the Nature of a NSC?” Section) that exhibit high self-renewing potential and generate transit amplifying progenitors that significantly increase the cell-generation capacity per initial NSC. Gyrenecephalia—mainly observed in primates—is also correlated with the addition of outer subventricular zone progenitors (Florio and Huttner, 2014). This evolutionary process of expansion of the embryonic NSC pool through diversification has not been cell-autonomous, with the embryonic microenvironment (the extracellular matrix, for example) playing a crucial role (Garcion et al., 2004; Loulier et al., 2009; Fietz et al., 2012; Pollen et al., 2015). However, only limited evidence exists to suggest a contribution of the vasculature. The chicken germinal cortical zones remain largely a-vascular (Rodriguez Celin et al., 2015) and in mice, even though BVs appear at the time of expansion of the neuroepithelial cell pool, only transit amplifying neural progenitors proliferate in close proximity to them (Vasudevan et al., 2008; Javaherian and Kriegstein, 2009). Nevertheless, in both examples the long basal processes of radial glia remain in constant contact with BVs positioned deeper in the tissue (Vasudevan et al., 2008; Rodriguez Celin et al., 2015) a feature shared by adult NSCs (Mirzadeh et al., 2008).

**Figure 2:**
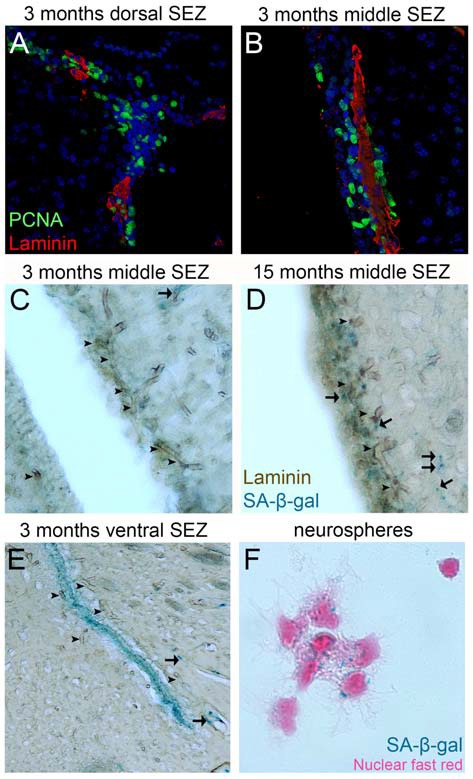
**Proliferation and senescence in the subependymal zone (SEZ). (A,B)** High magnification photographs of domains of the SEZ (dorsal in **A**, middle in **B**) taken from young adult mouse brain tissue immunostained for PCNA (to mark proliferating cells) and laminin (to mark blood vessels, BVs). Note the existence of multiple proliferating cells around the long BV running in parallel to the lateral ventricle in **(B)**, but also the existence of high proliferative activity in areas distant from BVs in **(A)**. **(C–E)** High magnification photographs of domains of the SEZ (middle in **C,D** and ventral in **E**) taken from young (in **C,E**) and aged (in **D**) rat brain tissue immunostained for laminin and chemically stained for senescence-associated β gal (in blue). Arrowheads indicate BVs and arrows senescent cells. Note the significantly lower density of BVs at the non-neurogenic side of the lateral ventricle (at the left of **C**), the existence of senescent cells along BVs outside the SEZ and the existence of high numbers of senescent cells in the ventral domain of the young-adult rat SEZ (in **E**). **(F)** High magnification of adult mouse NSCs isolated from the SEZ and kept in culture. Note the existence of senescent cells (nuclei are counterstained with nuclear fast red). [Antibodies used: rabbit anti-laminin: 1/500 (Abcam), mouse anti-PCNA: 1/500 (Abcam). Alexa goat anti-rabbit 568 and goat anti-mouse 488 (Invitrogen). Biotinylated goat anti-rabbit and DAB staining kit (Vector laboratories). Senescence-associated β gal staining kit (Millipore). Adult NSCs cultured in DMEM/F12 supplemented with B27 (Gibco), FGF2 (20 ng/ml) and EGF (20 ng/ml). All animal work was performed in accordance with the UK Animals (Scientific Procedures) Act 1986 and was approved by the University of Cambridge Animal Welfare and Ethical Review Body].

In the postnatal mammalian brain active NSCs survive in specialized NSC niches (Kazanis, 2013) and accumulating evidence points to the vasculature as an important element of these microenvironments (Goldman and Chen, 2011). The BV bed in the NSC niche of the subependymal zone (SEZ-located at the lateral walls of the lateral ventricles) is different from all neighboring areas: the density of BVs is higher and vessels are positioned differently in respect to the plane of the ventricle (Figures 2C,E; Kazanis et al., 2010; Culver et al., 2013). Furthermore, BVs are more leaky (Tavazoie et al., 2008) and blood flow is slower, suggesting the existence of hypoxic conditions (Culver et al., 2013). This finding is consistent with reports revealing enhanced efficiency in culturing neural progenitors under hypoxia (Stacpoole et al., 2013). On the other hand, we have shown that in evolution—for example, when comparing rodent brains of different sizes—the number of adult NSCs that populate the niche correlates strictly with the number of ependymal cells and not with the volume of the niche that would reflect the volume of the vasculature (Kazanis and ffrench-Constant, 2012). This strengthens the hypothesis that during evolution/development the role of the vasculature becomes crucial for NSCs after they have been established in the system. Adult NSCs remain in a stage of quiescence (Doetsch et al., 1999). We have shown that NSCs are preferentially positioned next to the ependyma (Kazanis et al., 2010; Kazanis and ffrench-Constant, 2012), which produces pro-neurogenic signals such as noggin (Lim et al., 2000). However, recent experimental work revealed that NSC quiescence is controlled via direct cell-to-cell contacts with endothelial cells (Ottone et al., 2014) and via the activity of diffusible endothelium-derived factors such as neurotrophin-3, angiopoietins 1 and 2 and placental growth factor 2 (PlGF-2; Masjkur et al., 2012; Delgado et al., 2014; Crouch et al., 2015; see also reviews of Goldman and Chen, 2011; Ottone and Parrinello, 2015). In contrast, the mitotically active transit amplifying progenitors are physically located in close proximity to BVs and specifically in domains void of astrocytic endfeet and pericytes (Mirzadeh et al., 2008; Shen et al., 2008; Tavazoie et al., 2008). However, our work also indicates that the proximity to BVs cannot be the only factor controlling adult neural progenitor activity because within the narrow architecture of the niche mitotic cells are often positioned only at the side of BVs facing the lateral ventricle (Kazanis et al., 2010) while numerous proliferating progenitors can be also found away from BVs (Figures 2A,B).

In homeostatic conditions the majority of cells generated in adult niches die via apoptosis (Morshead and van der Kooy, 1992; Morshead et al., 1998). An alternative pathway is senescence: the exit from the cell cycle without differentiation. Senescence has not been properly investigated in adult NSCs, with the exception of one report on oligodendrocyte progenitor cells entering senescence during ageing (Kujuro et al., 2010). We and others have observed that a small fraction of adult NSCs show signs of senescence when cultured *in vitro* (Figure 2; Ross et al., 2008). We have also reported that senescent cells can be detected in the ventral domain of the SEZ even in young adult rats (Kazanis et al., 2013) and that in the same area normal mitotic activity and response of NSCs to injury are significantly weaker when compared to dorsal domains (Kazanis et al., 2013). Furthermore, the occurrence of senescent cells spreads dorsally over time (Figure 2), a phenomenon that seems to correlate with the gradual age-related shrinkage of the SEZ (Shook et al., 2012). So far there is no evidence that the BV network shows significant structural or functional variation among different domains of the niche (for example, in the ventral areas) or that this might be crucial in the occurrence of senescence. However, the observation that mitotically active progenitors are located proximal to BVs can lead to the hypothesis that remoteness from BVs might be correlated with senescence, or even cell death of NSCs.

### Commitment and Differentiation

It could be hypothesized that as the brain became larger and more complicated during evolution, the contribution of BVs in controlling the commitment and differentiation of NSC increased. Interestingly, similarly to diversification, there is no strong evidence on the existence of such dependance. The single example of a vessel-derived factor regulating cell fate decisions is pigment epithelium-derived factor (PEDF) that acts to instruct adult NSCs of the SEZ to switch mode of division from asymmetric differentiating to self-renewing (Ramírez-Castillejo et al., 2006). More specific to differentiation is the role of PlGF-2, diffused from endothelial cells and pericytes, that was recently shown to bias cell fate of adult NSCs and transit amplifying progenitors towards neurogenesis, at the expense of astrogliogenesis (Crouch et al., 2015). Enhanced neurogenesis was also observed in co-cultures of human NSCs and endothelial cells; albeit via unknown mechanisms (Chou et al., 2014). The available evidence suggests that cell fate choices are primarily controlled in a cell-autonomous manner, as has been shown from *in vitro* cultures of isolated embryonic and adult NSCs (Okano and Temple, 2009; Ortega et al., 2013). It should be noted that BV-derived signals might not be essential for instructing cell-fate of neural stem and progenitor cells, but for the survival of certain types of newborn neurons (Kirschenbaum and Goldman, 1995; Leventhal et al., 1999), a “selection” role that can give the illusion of an effect on cell-fate instruction.

### Migration

During embryonic development neural stem and progenitor cells migrate using radial glial processes, while adult SEZ-derived neuroblasts use chain-migration to exit the niche and reach their target area via an extracellular matrix-rich corridor, the rostral migratory stream. The first solid evidence that BVs also play a role in migration of neural progenitors came from animal models of cerebral ischemia, in which neuroblasts were shown to migrate towards the area of infarction along BVs (Yamashita et al., 2006; Thored et al., 2007; Kojima et al., 2010). Subsequently, vessel-supported migration was also found to be part of the homeostatic movement of neural progenitors, either in the granular cell layer in the hippocampus (Sun et al., 2015), or along the rostral migratory stream (Bovetti et al., 2007) and within the olfactory bulbs (Bovetti et al., 2007). Even more recently, BVs were shown to facilitate migration of oligodendroglial progenitors from the SEZ to the corpus callosum (Cayre et al., 2013) and the invasion of glioblastoma tumour cells into neighboring areas of the brain (Dubois et al., 2014). In the SEZ, SDF1/CXCL12 acts to attract neuroblasts expressing the CXCR4 receptor toward BVs (Kokovay et al., 2010), while endothelial-derived BDNF has been implicated to the attraction of neuroblasts to ischemic areas (Grade et al., 2013) and netrin-1 is necessary for the migration of oligodendroglial progenitors to the corpus callosum (Cayre et al., 2013). Overall, accumulating evidence indicates that migration is one phase in the life of NSCs that is highly dependent on the vasculature.

## Coupling of Neurogenesis and Angiogenesis; Who Needs Whom?

A strong correlation between angiogenic and neurogenic events has been observed but the functional substrate remains elusive; hence, the term “coupling” has been adopted. Such coupling events have been described in the developing rodent nervous system, in which endothelial cells share expression of transcription factors with surrounding NSCs according to the anatomical location (Vasudevan et al., 2008) and in the plastic areas of the adult song-bird. Moreover, in the post-stroke recovery in the adult rodent brain, induction of angiogenesis and of NSC-driven cytogenesis seem to be co-ordinated (Thored et al., 2007; Plane et al., 2010; Zhang et al., 2014), while pulses of synchronous NSC proliferation in the SEZ induce increased blood flow (Lacar et al., 2012a). Although some architectural and structural specializations of the adult NSC niche vasculature have been described, leading to the use of the term “neurovascular” niche, the absence of a functional specialization in BVs outside the niche has not yet been proven. Recent experimental work demonstrated that isolated endothelial cells from non-neurogenic areas of the adult brain exhibit equal, if not superior, potential in promoting NSC proliferation and differentiation when compared to endothelial cells from neurogenic areas (Crouch et al., 2015). In addition, mitotically active oligodendrocyte progenitors in the brain parenchyma have been reported to cross-talk with endothelial cells within “oligovascular niches” (Arai and Lo, 2009; Pham et al., 2012). Notably, in experimental animal models of stroke or multiple sclerosis, transplanted NSCs form “atypical neurovascular niches” using BVs outside the established stem cell areas (Pluchino et al., 2010). Finally, accumulating evidence suggests that dormant NSCs exist in the non-neurogenic brain parenchyma of rodents (Sirko et al., 2013), possibly next to BVs (Bardehle et al., 2013), and that in the human brain such progenitors might not be dormant at all (Ernst et al., 2014). On the other hand, we have observed that, in response to a demyelinating lesion in the corpus callosum, platelets accumulate specifically in the vasculature of the SEZ, suggesting an underlying specialization of BVs (Kazanis et al., 2015). Furthermore, by staining for senescence-associated markers we have also observed that although a high number of endothelial cells in BVs spread throughout the adult rat brain are senescent, the niche vasculature remains senescence-free (Figure 2), a possible indication of higher vascular plasticity potential in the specific area.

Another significant aspect of the cross-talk between BVs and NSCs is the transport of factors that might be important to NSCs but are not produced by vascular and perivascular cells. Two examples have already been mentioned: oxygen (Lacar et al., 2012a; Culver et al., 2013) and insulin (Masjkur et al., 2012). Recent experimental work has also revealed that blood-derived factors, such as GDF11, can act to rejuvenate aged neural stem and progenitor cells (Ruckh et al., 2012; Katsimpardi et al., 2014). In other words, by feeding the aged brain with young blood, scientists were able to reverse some of the effects of ageing on NSCs. However, it still remains unknown if the effect was direct to NSCs or if it was dependent on rejuvenating the vasculature or macrophages. A final and intriguing aspect is the possible instructive role of NSCs on the vasculature. The dominant hypothesis is that the vasculature directs NSCs, exemplified by the observation that grafted NSCs are ectopically homed perivascularly (Pluchino et al., 2010). However, SEZ NSCs can influence the function of BVs, for example, the blood flow (Lacar et al., 2012b), and oligodendrocyte progenitors can control angiogenesis through hypoxia-inducible factors (Yuen et al., 2014). These are in concert with evidence that embryonic cortical NSCs are important for the establishment of the developing vasculature (Gerhardt et al., 2004; Ma et al., 2013) and that in many cases vascularization tightly follows the maturation of the nervous system (Rodriguez Celin et al., 2015). Recently published work with human NSCs also showed that they provide the necessary juxtacrine and paracrine signals to drive human endothelial cells to form “vasculature-like structures” (Chou et al., 2014) and promote angiogenesis in the rodent brain (Hicks et al., 2013). Therefore, the bidirectional cross-talk between NSCs and BVs is a line of research that needs to be developed further.

## Author Contributions

CK contributed original data wrote and approved the manuscript. IK contributed original data, developed the concept, wrote and approved the manuscript.

## Funding

IK was supported by Action Medical Research, UK (GN2291).

## Conflict of Interest Statement

The authors declare that the research was conducted in the absence of any commercial or financial relationships that could be construed as a potential conflict of interest.
